# Endo-1,4-β-D-xylanase Assay Using Azo-Xylan and Variants Thereof

**DOI:** 10.21769/BioProtoc.5283

**Published:** 2025-04-20

**Authors:** Luca Bombardi, Annalaura Coltro, Salvatore Fusco

**Affiliations:** Biochemistry and Industrial Biotechnology (BIB) Laboratory, Department of Biotechnology, University of Verona, Verona, Italy

**Keywords:** Xylanases, Enzymatic assay, Azo-Xylan, Lignocellulosic biomass, Dyed xylan, Thermostability, Colorimetric assay, Xylan degradation

## Abstract

Xylan is the main component of hemicellulose and consists of a complex heteropolysaccharide with a heterogeneous structure. This framework, in addition to the crystalline structure of cellulosic fibers and the rigidity of lignin, makes lignocellulosic biomass (LCB) highly recalcitrant to degradation. Xylanases are glycoside hydrolases that cleave the β-1,4-glycoside linkages in the xylan backbone and have attracted increasing attention due to their potential uses in various industrial sectors such as pulp and paper, baking, pharmaceuticals, and lignocellulosic biorefining. For decades, the measurement of xylanase activity was based on reducing sugar quantification methods like DNS or Nelson/Somogyi assays, with numerous limitations in terms of specificity and interference from other enzymatic activities. A better alternative is the colorimetric Azo-Xylan assay, which specifically measures the endo-1,4-β-D-xylanase activity. In this study, the Azo-Xylan protocol was adapted from the company Megazyme to determine the enzymatic activity of thermostable xylanases produced by microbial consortia (i.e., microbiomes), aiming to determine biochemical features such as temperature and pH optima, thermostability, and shelf life. This modified approach offers a rapid, cost-effective, and highly specific method for the determination of xylanase activity in complex mixtures, helping the development of a xylanase-based method for the hydrolysis of hard-degrading substrates in bio-based industries.

Key features

• Direct enzyme assay for qualitative xylanase activity detection or quantitative measurement with a calibration curve.

• Specific for determination of endo-1,4-β-D-xylanase activity, allowing to overcome interferences by enzymes with other activities.

## Graphical overview


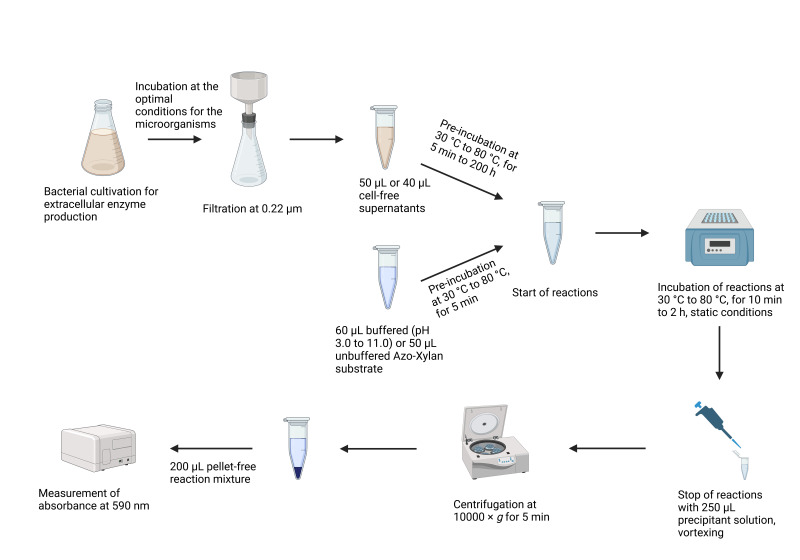


**Key steps of the Azo-Xylan assay**

## Background

Xylan is one of the most abundant polysaccharides in nature, having a highly complex tertiary structure organized as a linear backbone of D-xylose with β-1,4-linkages, usually branched with other sugars such as α-arabinose and/or organic acids [1,2]. Due to its heterogeneous architecture, xylan degradation is very challenging, but the process can be effectively carried out by xylanases that hydrolyze β-1,4-glycosidic bonds. Xylanases are glycoside hydrolases (GHs) expressed by various organisms, including bacteria, yeast, fungi, and crustaceans [3]. The genomic, structural, and functional information of xylanase can be found in the carbohydrate-active enzyme database (CAZy) [4]. Xylanases are generally associated with GH families 5, 7, 8, 9, 10, 11, 12, 16, 26, 30, 43, 44, 51, and 62 [4], hydrolyzing xylan substrates according to two modes of action: retention and inversion [5,6]. This wide spectrum of physicochemical properties, substrate specificities, and catalytic mechanisms makes xylanases highly versatile for numerous applications across industrial and biotechnological sectors [7]. Nowadays, their use spans pulp bleaching in pulp and paper industries [8], bakery and food processing industries [9,10], prebiotics and potential anticancer agents in pharmaceutical sectors [11,12], and lignocellulosic biorefining [13].

**Figure 1.**
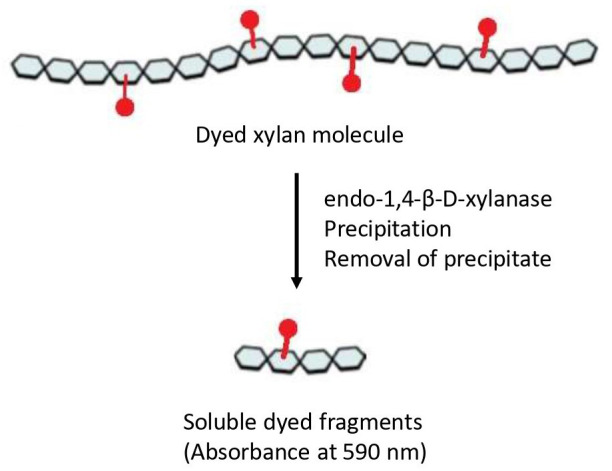
Representation of the Azo-Xylan molecule. Adapted from the picture available at Megazyme website (https://prod-media.megazyme.com/media/pdf/71/fd/ce/enzyme-substrates-brochure.pdf).

Numerous methods have been developed to measure endo-xylanase activity. These assays differ not only in assay conditions (temperature, incubation time, substrate, etc.) but also in the quantification method of enzymatic activity. For decades, the preferred method was the measurement of reducing sugars liberated from the polymer through the 3,5-dinitrosalicylic acid (DNS) method [14] or the Nelson/Somogyi method [15,16]. However, both procedures present some limitations, especially in terms of a lack of stoichiometric relation of the color response and turbidity [17]; in addition, these assays are nonspecific, and other enzymatic activities can be involved in the product determination, such as β-xylosidase and α-glucuronidase [18]. Viscosimetric methods provide benefits in terms of specificity and sensitivity, but they are also time-consuming and show a limited throughput of just a few reactions per day [18]. Nevertheless, fermentation broths or complex mixtures are generally composed of a cocktail of endo-xylanase and other glycosidases, including α-L-arabinofuranosidase and β-xylosidase, making the process of quantification quite challenging. To overcome these limitations, soluble dyed polysaccharides, such as Azo-Xylan or Azo-wheat arabinoxylan, are extensively used and give the possibility to measure enzymatic activity even in preparations with high levels of reducing sugars [18]. The Azo-Xylan assay is specific for endo-1,4-β-D-xylanase activity, and the protocol can be carried out following the manufacturer’s instructions (Megazyme, Bray, Ireland). In principle, to obtain Azo-Xylan, birchwood xylan is first purified to remove starch and then it is chemically dyed with Remazol Brilliant Blue R™ to an extent of approximately one dye molecule per 30 sugar residues. After incubation of Azo-Xylan with endo-xylanases, the substrate is depolymerized to produce low-molecular-weight water-soluble fragments (Figure 1), which remain in solution, resulting in a colored supernatant that can be directly correlated to endo-xylanase activity by reference to a standard curve (Megazyme, Bray, Ireland). Herein, we adapted the classical version of this protocol to measure the endo-xylanase activity of thermostable xylanases secreted by different microbial consortia, aiming to the determination of enzyme temperature and pH optima, thermostability, and shelf life [19,20]. The modifications can help in the determination of the biochemical parameters of enzymes in complex mixtures and can enhance the possibility of obtaining a complete characterization in a rapid and cheap way.

## Materials and reagents

**Biological materials**

1. Cell-free supernatants (CFSs), mixture of extracellularly secreted enzymes obtained from filtration of microbial culture broth at 0.22 μm

**Reagents**

1. Azo-Xylan from Birchwood (Megazyme, catalog number: S-AXBP)

3. Ethanol (Honeywell, catalog number: 02870)

4. Citric acid (Merck, catalog number: 1.00244.1000)

5. Sodium dihydrogen phosphate (NaH_2_PO_4_) (Chemlab, catalog number: CL00.1497.1000)

6. Disodium hydrogen phosphate (Na_2_HPO_4_) (Chemlab, catalog number: CL00.1463.1000)

7. Potassium dihydrogen phosphate (KH_2_PO_4_) (Sigma-Aldrich, catalog number: P5655)

8. Tris (Neofroxx, catalog number: 1125KG001)

9. CAPS (Sigma-Aldrich, catalog number: C2632)

10. Hydrochloric acid (HCl) (Honeywell, catalog number: 30721)

11. Sodium hydroxide (NaOH) (Honeywell, catalog number: 30620)

**Solutions**

1. Azo-Xylan substrate (see Recipes)

2. Citrate buffer (see Recipes)

3. Sodium phosphate buffer pH 6.0 (see Recipes)

4. Sodium phosphate buffer pH 7.0 (see Recipes)

5. Sodium phosphate buffer pH 8.0 (see Recipes)

6. Potassium phosphate buffer (see Recipes)

7. Tris-HCl buffer (see Recipes)

8. CAPS buffer (see Recipes)

9. Precipitant solution (see Recipes)

**Recipes**

**1. Azo-Xylan substrate (100 mL)**

**Table:** 

Reagent	Final concentration	Quantity or Volume
Azo-Xylan powder	1% w/v	1 g
Distilled water (dH_2_O)	n/a	to 100 mL, see note
Total	1% w/v	100 mL

*Note: The Azo-Xylan powder should be added to 80 mL of boiling distilled water under vigorous stirring on a hot plate stirrer. Turn off the heat and continue stirring until the suspension has mixed completely. Add water to 100 mL.*

**2. Citrate buffer (100 mL)**

**Table:** 

Reagent	Final concentration	Quantity or Volume
Citric acid	2.63 M	9.60 g
HCl (6 N)	n/a	to pH 3.0, 4.0, or 5.0
dH_2_O	n/a	to 100 mL, see note
Total	n/a	100 mL

*Note: Dissolve the citric acid in 80 mL of dH_2_O. Adjust the pH to the desired value. Add water to 100 mL.*

**3. Sodium phosphate buffer pH 6.0 (100 mL)**

**Table:** 

Reagent	Final concentration	Quantity or Volume
NaH_2_PO_4_ solution (1 M)	438 mM	43.80 mL
Na_2_HPO_4_ solution (1 M)	62 mM	6.20 mL
dH_2_O	n/a	to 100 mL
Total	n/a	100 mL

**4. Sodium phosphate buffer pH 7.0 (100 mL)**

**Table:** 

Reagent	Final concentration	Quantity or Volume
NaH_2_PO_4 _solution (1 M)	195 mM	19.50 mL
Na_2_HPO_4_ solution (1 M)	305 mM	30.50 mL
dH_2_O	n/a	to 100 mL
Total	n/a	100 mL

**5. Sodium phosphate buffer pH 8.0 (100 mL)**

**Table:** 

Reagent	Final concentration	Quantity or Volume
NaH_2_PO_4_ solution (1 M)	34 mM	3.40 mL
Na_2_HPO_4_ solution (1 M)	466 mM	46.60 mL
dH_2_O	n/a	to 100 mL
Total	n/a	100 mL

**6. Potassium phosphate buffer (100 mL)**

**Table:** 

Reagent	Final concentration	Quantity or Volume
KH_2_PO_4_	0.50 M	6.80 g
HCl (6 N)	n/a	to pH 6.0 or 7.0
dH_2_O	n/a	to 100 mL, see note
Total	n/a	100 mL

*Note: Dissolve KH_2_PO_4_ in 80 mL of dH_2_O. Adjust the pH to the desired value. Add water to 100 mL.*

**7. Tris-HCl buffer (100 mL)**

**Table:** 

Reagent	Final concentration	Quantity or Volume
Tris-base powder	0.50 M	6.06 g
HCl (6 N)	n/a	to pH 7.0, 8.0 or 9.0
dH_2_O	n/a	to 100 mL, see note
Total	n/a	100 mL

*Note: Dissolve the Tris-base powder in 80 mL of dH_2_O. Adjust the pH to the desired value. Add water to 100 mL.*

**8. CAPS buffer (100 mL)**

**Table:** 

Reagent	Final concentration	Quantity or Volume
CAPS powder	0.50 M	11.06 g
NaOH (5 M)	n/a	to pH 10.0, 11.0
dH_2_O	n/a	to 100 mL, see note 5
Total	n/a	100 mL

*Note: Dissolve the CAPS powder in 80 mL of dH_2_O. Adjust the pH to the desired value. Add water to 100 mL.*

**9. Precipitant solution (100 mL)**

**Table:** 

Reagent	Final concentration	Quantity or Volume
Ethanol 95%	95% v/v	100 mL
Total	95% v/v	100 mL

**Laboratory supplies**

1. 1.5 mL plastic tubes (Sarstedt, catalog number: 72.690.001)

2. 200 μL pipette tips (Manufacturer, catalog number: 70.3030.020)

3. 1000 μL pipette tips (Manufacturer, catalog number: 70.3050)

4. 96-well plates (Sarstedt, catalog number: 83.3924.500)

## Equipment

1. LLG-uniTHERMIX Pro thermo shaker (Lab Logistics Group GmbH, catalog number: 6.263 484)

2. Centrifuge (Eppendorf, model: 5430 R)

3. BioTek Synergy Neo2 hybrid multi-mode reader (Agilent Technologies, model: BTNEO2)

## Software and datasets

1. Gen5^TM^ Microplate Reader and Imager Software (BioTek Instruments, Inc., version 3.10)

2. GraphPad Prism 9

3. Excel

## Procedure

**A. Standard procedure**

1. Pre-incubation

a. Place 50 μL of 1% w/v Azo-Xylan substrate and 200 µL of each cell-free supernatant (CFS) in separate 1.5 mL plastic tubes.

b. Prewarm samples in a thermo shaker for 5 min at 50 °C under static conditions.

2. Start of reactions: mix 50 µL of each prewarmed CFS with 50 µL of prewarmed Azo-Xylan substrate.

3. Incubation: Incubate for 2 h at 50 °C under static conditions.

4. End of reactions

a. Add 250 µL of precipitant solution to each plastic tube.

b. Vortex the tubes.

c. Place at room temperature for 10 min.

**Figure 2.**
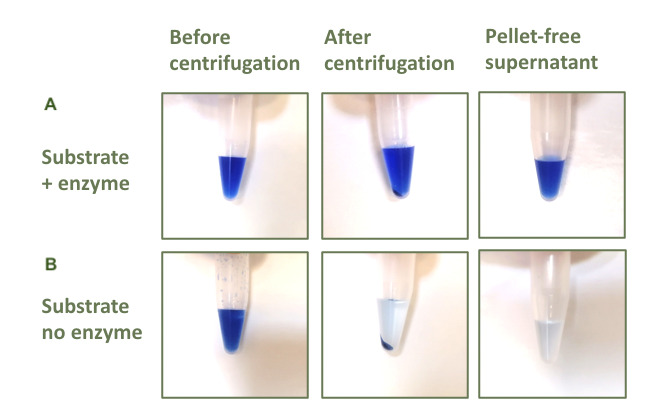
Reactions in tubes throughout the assay phases. (A) Reactions carried out incubating Azo-Xylan substrate and cell-free supernatants (CFSs). (B) Negative controls prepared by adding precipitant solution to the Azo-Xylan substrate before adding CFSs.

5. Clarification: Centrifuge at 10,000× *g* for 5 min (Figure 2).

6. Absorbance measurement

a. Transfer 200 µL of each clarified reaction mixture into a well of a 96-well plate.

b. Measure the absorbance at 590 nm.

**B. Variations on the standard procedure**

**Figure 3.**
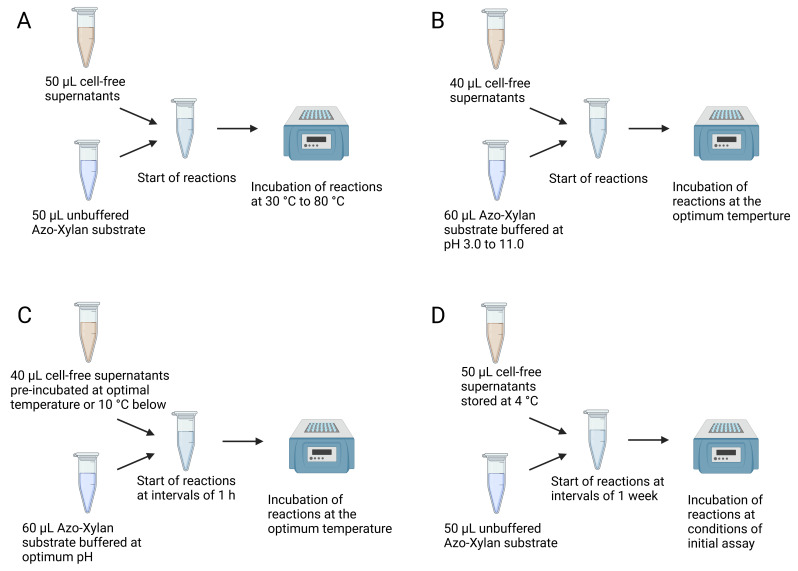
Summary of variations to the standard protocol. (A) Determination of optimum temperature. (B) Determination of optimum pH. (C) Determination of enzymatic thermostability. (D) Determination of enzymatic storage stability.

1. Determination of the optimal temperature (Figure 3A)

a. Carry out sample pre-incubations and reaction incubations at different temperatures.

2. Determination of the optimal pH (Figure 3B)

a. Add 10 µL of each buffer solution to tubes with 50 µL of Azo-Xylan substrate.

b. Pre-incubate substrates and samples at the optimum temperature.

c. Start reactions by adding 40 µL of each prewarmed CFS to the tubes with the buffered substrate.

d. Incubate for 1 h at the optimum temperature.

3. Determination of enzyme thermostability (Figure 3C)

a. Pre-incubate CFS aliquots at their optimum temperature and at 5 or 10 °C below their optimum.

b. Withdraw 40 µL aliquots at different time points.

c. Carry out assays at the optimum pH and temperature with 1 h incubation time.

4. Determination of enzyme storage stability (Figure 3D)

a. Periodically test CFSs stored at 4 °C via an assay with the same pH, temperature, and incubation time as previous assays for comparison.

**Critical points:** When handling multiple reaction tubes, make sure to stop the reactions in the same order they were started and with the same time interval between one tube and the next one to have the same incubation time for all tubes.

## Data analysis

Calculate the mean and standard deviation of absorbance values between technical triplicates. A template for the determination of temperature and pH optima, thermostability, and shelf life is available as an Excel file in Supplementary information.

## Validation of protocol

All experiments were run in technical triplicates of each biological triplicate. Negative controls were prepared by mixing 250 µL of the precipitant solution with 50 µL of Azo-Xylan (with 10 µL of buffer solution in the case of buffered assays) before adding 50 µL of CFS (or 40 µL in the case of buffered assays).

This protocol or parts of it has been used and validated in the following research articles:

- Bombardi et al. [20]. Lignocellulolytic Potential of Microbial Consortia Isolated from a Local Biogas Plant: The Case of Thermostable Xylanases Secreted by Mesophilic Bacteria. Int J Mol Sci.
- Bombardi et al. [19]. Thermophilic Hemicellulases Secreted by Microbial Consortia Selected from an Anaerobic Digester. Int J Mol Sci.

## General notes and troubleshooting

**Troubleshooting**

Problem 1: The absorbance reading at 590 nm exceeds the linearity range of the spectrophotometer.

Possible cause: The enzymes in the CFSs are too concentrated.

Solution: Reduce the incubation time of the reactions.

Problem 2: The absorbance reading of negative controls is too high (>0.1).

Possible causes: The Azo-Xylan polymer has degraded over time, perhaps because of contamination with xylanases.

Solution: Prepare a fresh Azo-Xylan suspension every 2–3 months and store it at 4 °C when not in use. Cover the bottle with aluminum foil for protection from light.

Problem 3: The volume of liquid in the vials at the end of the incubation time is lower than expected.

Possible cause: The incubation temperature caused evaporation of some of the liquid.

Solution: At the end of the incubation time, place the vials on ice for a few seconds before opening them to add the precipitant solution.

## Supplementary information

The following supporting information can be downloaded here:

1. Excel file: Data analysis template
